# Modeling the nexus between coal consumption, FDI inflow and economic expansion: does industrialization matter in South Africa?

**DOI:** 10.1007/s11356-020-07691-x

**Published:** 2020-01-15

**Authors:** Joshua Udi, Festus Victor Bekun, Festus Fatai Adedoyin

**Affiliations:** 1 Department of Economic, Federal University Lokoja, Lokoja, Kogi State Nigeria; 2 Faculty of Economics Administrative and Social sciences, Istanbul Gelisim University, Istanbul, Turkey; 3 Department of Accounting, Finance and Economics, Bournemouth University, Poole, England UK

**Keywords:** Coal consumption, FDI inflow, Economic expansion, Industrialization, South Africa

## Abstract

This study examines the role of industrialization in the energy-growth-FDI nexus for the case of South Africa using data over the period 1970 to 2018. The empirical exercise was conducted using Pesaran Autoregressive Distributed Lag (ARDL) bounds testing approach. To accomplish our study objective, we analyze stationarity properties of the series using the unit root test after which we applied Bayer-Hanck (B-H) combined technique to cointegration to assess whether a long-run relationship exists among the series. Empirical results show that a 1% change in FDI account for 0.002% and 0.013% increase in economic expansion in the short- and long- run respectively. Also, a 1% increase in coal consumption influence GDP negatively by 0.083% and 0.207% in the short and long run respectively. Furthermore, a 1% increase in total natural resource rent positively affects GDP by 0.02% and 0.05% respectively in the short and long run. Industrialization, on the other hand, demonstrates a positive and significant impact on the economic growth process both in the short and long run. Industrialization contributes 0.506% and 1.274% to economic expansion both in the short and long run respectively. The causality tests suggest that a one-way causal link running from FDI to industrialization and from industrialization to coal consumption exists. Finally, FDI inflow drives total natural resource rents in South Africa. This study also gives reliable growth and energy policy proposals to policymakers applicable to countries around the globe.

## Introduction

The linkage between foreign direct investment (FDI hereafter) inflow and economic prosperity whether positive or otherwise has been an issue of increasing concern. While some studies support the nexus, others disagree. Others prove neutrality between the series in question. For instance, recent studies (Kalai and Zghidi 2019; Sokhanvar 2019; Sarkodie and Strezov 2019) confirmed the said nexus, while other authors such as the studies of Zandile and Phiri (2019), Nyoni (2018), Abdouli and Hammami (2017), and Herzer (2012) questioned the FDI-growth nexus. The case presented above is similar to the South African scenario. Despite the existing volume of research on FDI-growth nexus, it is imperative to point out clearly that FDI that flow into the South Africa economy has increased drastically over the last three decades which demand the need for further empirical investigation on the nexus.

Furthermore, South Africa’s economy has on average been the gateway of an inward foreign investment that flows into the Southern African region. For example, in 2010, the country achieved an inward FDI of $1.23 billion which sharply increase to $5.81 billion in 2011 UNCTAD (2012). This placed the country as the largest recipient in the region as well as the second biggest in the continent behind leader Nigeria. In 2017, the FDI inflow to South Africa reversed to $2.0 billion UNCTAD (2018). The report further states that in 2018 the FDI inflow to the southern region experienced an increase by 13% to $32 billion out of which South Africa achieved the largest shares of about $5.3 which indicates a sharp increase compared with 2017. Note that initially, statistic indicates that the major investors of FDI in South Africa are UK economies (87%), Germany (6%), Asia (2.3%), developing economies (4%) (UNCTAD 2013). However, a consensus has not been established yet as regards the reality of the impact of FDI on economic growth. Prominent among the previous studies especially the most recent ones are Khobai et al. (2017) and Tshepo (2014) for South Africa.

Thus, this study stands out among other studies in term of scope as industrialization is incorporated in the functional model as a control variable for the first time in the case of South Africa. This became necessary because of two reasons: firstly, a report that South Africa is the largest industrialized economy in the Southern African region as well as the second largest in the continent after Egypt World Bank economic indicator (WDI 2017). Secondly, attracting FDI is in part dependent on the conditions of the local firms as stress by the studies of Nunnenkamp and Spatz (2003) and Singh and Jun (1999). Therefore, this study sets out to achieve the followings: first, to investigate the causal effect of FDI on economic growth and to investigate the long-run relationship between the series. Second, to ascertain whether or not the claim made by Nunnenkamp and Spatz (2003) and Singh and Jun (1999) is a reality in South Africa. In addition to the above, the study set out to investigate the growth hypothesis which claims that coal consumption is a driver of economic prosperity.

Moreover, the current study strongly believes that this debate is still subject to a single country investigation. This claim is supported by the assertion made by Shahbaz et al. (2013) who submit that the argument surrounding the coal-led growth hypothesis is still very much an ongoing one particularly for a single country. The reason is that the previous studies are flooded with conflicting outcomes. For instance, studies (such as Bekun et al. 2019a, b; Saint Akadiri et al. 2019) supported the growth hypothesis for the South Africa economy consistent with Wang et al. (2018), Sarkodie and Adams (2018), and Ulucak and Bilgili (2018). Other previous studies hold an inverse view claiming that economic expansion is the reason for the demand of coal usage (see Álvarez-Herránz et al. 2017; Govindaraju and Tang 2013; Jinke et al. 2008; Wolde-Rufael 2010). Conclusively, this study went one more step further to include total natural resources rent as an additional variable to the model framework. The reason is no far-fetched from the fact that the South Africa economy will hardly survive without the natural sources.

The rest of the paper consist of section 2 review relevant literatures explaining the FDI-led growth hypothesis and the coal-led growth nexus couple with the various theories upon which the this study stands, while section 3 talks about the methodology and the functional model, the result from the findings are interpreted in section 4, and finally section 5 takes care of the concluding remarks and policy direction.

## Review of literature

**Fig. 1:**
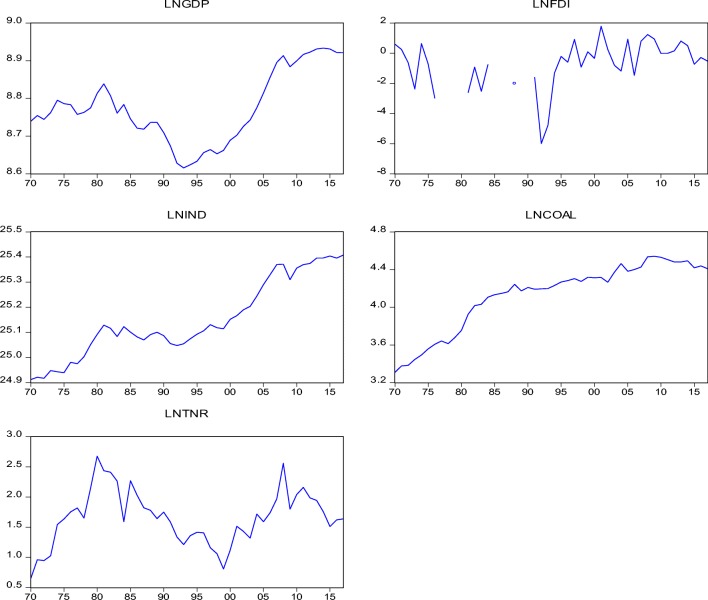
Visual of the variables of study

Empirically, the FDI-led growth hypothesis is still very much subject to empirical verification as consensus is yet to be established for conclusion. While one side supports the acclaimed nexus, other opposes its reality. For instance, a recent study (Kalai and Zghidi 2019) carried on MENA economies using the dynamic Autoregressive Distributed Lag (ARDL) summarily confirmed a one-way causal link running from FDI inflow to economic expansion of the MENA economies. The finding of the study went further to establish long-run co-movement of the series of investigation. In a related study, Sokhanvar (2019) investigates the said hypothesis for the European Union economies which are significant recipient of FDI inflow. The result from the finding proves that FDI inflow and tourism are determinant factor for economic expansion of the region. The study went further to adopt the impulse responses response function to complement the block exogeneity Wald test which revealed that the impact of FDI inflow is a mirage as the finding indicates a negative relationship between the variables (Fig. 1).

Furthermore, Sarkodie and Strezov (2019) studied the effect of FDI inflow, economic expansion, and energy consumption on greenhouse gas emission in the emerging economies. The finding confirmed the positive impact of only a clean FDI inflow that is free from posing environmental danger. In the case of South Africa, Sunde (2017) carried out a research on the relation between FDI inflow, export, and economic expansion using an ARDL approach. His findings show that both FDI inflow and export induced economic expansion. The granger causality test also indicates a one-way flow of causal effect running only from FDI inflow to economic progress. The study of Borensztein et al. (1998) submits that the impact of FDI inflow on economic growth is favorable though dependent on the absorptive capacity of the host country. He further claimed that once a country achieved at least the threshold level, FDI inflow will natural spur economic acceleration. This is similar to the work of Gungor and Katircioglu (2010), Güngör and Ringim (2017), Güngör et al. (2014), and Tshepo (2014). The work of Nistor (2014) confirmed the FDI and economic progress association for Romania.

Abbes et al. (2015) and Omri and Kahoulib (2013) conducted a panel research comprising of 65 countries where the overall outcome indicates that FDI inflow is a key promoter of economic growth confirming the work of Tang et al. (2008), Malikane and Chitambara (2017), Zghidi et al. (2016), Nasrin and Khan (2016), Almfraji and Almsafir (2014), Inekwe (2013), and Ayadi (2010). Additionally, Adams (2009) submits that the relationship between FDI and economic growth is positive only in the long run. Abdouli and Hammami (2017) found a country-based rather general impact of FDI inflow on economic growth. Their findings confirm the FDI-growth nexus for the region of their interest with the exclusion of Egypt and Lebanon. This is similar to the work of Abdouli and Hammami (2017) in the case of the MENA countries. The work of Srinivasan et al. (2011) reveals a bidirectional link between FDI and economic growth for most of the SAARC countries with the exclusion of India where a unidirectional link was found. Their findings validate the works of Lee (2013).

Similarly, Shahbaz and Rahman (2012) investigated the effect of financial development, import, and FDI inflow in Pakistan, and found a cointegration between the variables. The study further asserts that all the variables with FDI inflow inclusive exert positive impact on economic expansion. Claassen et al. (2011) and Carike et al. (2012) reveal evidence of a bidirectional link between FDI inflow and economic expansion. Secondly are the works of authors who question the FDI-growth nexus. For example, Nyoni (2018) found that the impact of FDI on economic expansion of Zimbabwe is a fallacy. Zandile and Phiri (2019) carried out research on the subject matter for Burkina Faso using ARDL bound test approach. The finding could not ascertain the impact of FDI inflow on economic expansion both directly and indirectly. Other studies with opposing view include Abdouli and Hammami (2017) and Herzer (2012) for the MENA countries which revealed a negative connection between FDI inflow and economic progress in Egypt and Lebanon. Adams (2009) shows a short-run negative impact of FDI on domestic investment. Other studies remain neutral as to whether or not FDI inflow drives economic growth (Flora and Agrawal 2014; Pandya and Sisombat 2017; Mehic et al. 2013). Goh et al. (2017) submit that on the overall, there is no evidence of the positive impact of FDI in the long run for the Asian economies, confirming the work of Mah (2010) and Khobai et al. (2017). Bezuidenhout (2009) proved that the perceived impact of FDI on economic growth is a fallacy for the southern Africa region.

Succinctly, the argument about the coal-led growth hypothesis is still ongoing because of the inability of the previous studied to establish concluding remark due to conflicting interest. Thus, there are studies that lent their view in support of the coal usage as a driver of economic expansion while others hold the opposite view. For example, among the studies that support this hypothesis is the work of Balsalobre-Lorente et al. (2018) who found that renewable electricity usage, natural resources, and energy innovation promoter of environmental quality extend enhances economic expansion in EU-5 economies. The study of Samir et al. (2018) ascertains that transport energy consumption contributes significantly to economic progress in the study region and that the transport infrastructure is of immense benefit to the economies.

Also, the study of Adedoyin et al. (2020) confirmed a two-way causal flow between energy usage and economic expansion and is consistent with the work of Shahbaz et al. (2013) (other supporting studies include Akadiri et al. 2019; Bekun et al. 2019a, b; Saint Akadiri et al. 2019; Wang et al. 2018; Sarkodie and Adams 2018; Ulucak and Bilgili 2018; Bekun et al. 2019a, b). Alvarez-Herranz et al. (2017) assert that at low-income level, increasing renewable energy sources will drive economic expansion accordingly. Secondly, some study advocates instead for conservative policy such Alvarez-Herranz et al. (2017) who assert that in the long run, increasing energy conservative policy will enhance environmental quality in the OECD economies and that energy innovation will become fully efficient only in the long term, similar to the work of Shahbaz et al. (2019). He further confirmed a feedback interaction between economic expansion and carbon emission and between FDI and carbon emission in the study area. Other study which claimed that the demand for coal is driven by economic expansion includes Govindaraju and Tang (2013), Jinke et al. (2008), Wolde-Rufael (2010), and Yuan et al. (2008). The study of Alola and Alola (2019) also confirmed a long-run relationship between the outlined variables under consideration.

### The theoretical link between FDI and economic growth

This study is premised on the widely known modernization and dependency theories to investigate the relation between FDI inflow and economic growth. Modernization theory asserts that the advancement of the economic process which is endogenous in nature is dependent on technological improvement and human capital development. According to this theory, the endogenous process required technological progress and human capital improvement which are the by-products of FDI inflow. This intuition is supported by the work of Pradhan (2002) and Li and Liu (2005).

Empirically, studies such as Kalai and Zghidi (2019), Sokhanvar (2019), and Sarkodie and Strezov (2019) lent their support to the modernization theory. They believe that FDI inflow comes with its spillover effect in form technological transfer; human capital development is a key to economic expansion in the recipient economy particularly the developing ones. On the other hand, the dependency theory sees FDI inflow as an exogenous factor that plays a key role in reversing economic expansion. This theory maintains that FDI inflow is a mere attempt for exploiting the transitory economies by the developed countries rather than rendering economic or political assistance as perceived.

Furthermore, the theory claims that FDI inflow is targeted at keeping the developing economies under perpetual poverty to forestall a presumed competition. Adams (2009) submits that FDI is a strong tool that facilitates crowding out effect on the host country investment, thus, a panacea for capital flight. Clark and Chan (1996) maintain that FDI normally leads to debt overhang for the developing countries as the creditor nations usually charge a very high-interest rate on external borrowing for which servicing normally deprive the domestic economy of the sources that would be injected into the productive sector of the economy. This claim is not without empirical back up (see Nyoni 2018; Zandile and Phiri 2019; Abdouli and Hammami 2017; Herzer 2012). They conclude in their respective studies that the impact of FDI inflow on economic expansion of the host nation is a mirage.

Similarly, there are four hypotheses that explain the relation between coal usage and economic acceleration. The first hypothesis, known as the growth hypothesis, described the positive influence of coal consumption on economic growth. According to this hypothesis, coal consumption induces economic expansion in a one-way direction. This proposition is backed by empirical studies (such as Akadiri et al. 2019; Bekun et al. 2019a, b; Saint Akadiri et al. 2019; Wang et al. 2018; Sarkodie and Adams 2018; Ulucak and Bilgili 2018; Bekun et al. 2019a, b). Bekun et al. (2019a, b) study the relationship between economic growth and coal consumption using the dynamic ARDL approach and found cointegration between the variables. They also confirmed that economic expansion is one of the consequences of coal usage in South Africa. Secondly, the conservation hypothesis holds the opposite view. This asserts that economic advancement induces the demand for coal consumption for power generation. This hypothesis maintained that coal consumption depend on the rate of economic expansion.

Álvarez-Herránz et al. (2017), Govindaraju and Tang (2013), Jinke et al. (2008), Wolde-Rufael (2010), and Yuan et al. (2008) established evidences in their respective studies in support of this hypothesis. On the other hand, the feedback hypothesis opines that there is a mutual interaction between coal consumption and economic progress which discourages the adoption of conservation policy as supported by the empirical work of Wolde-Rufael (2010), Yuan et al. (2008), and Yoo (2006). Finally, the neutrality hypothesis stressed that impact of coal consumption on economic growth could not be established, thus remained unaccountable. This proposition is also support by some empirical claims such as Wolde-Rufael (2009), Ziramba (2009), Jinke et al. (2008), and Fatai et al. (2004). According to them, the impact of coal consumption on economic progress is never felt.

## Data and methodology

This study intends to investigate the relationship between FDI inflow and economic expansion in South Africa using data within the time frame 1970 to 2018. The variables of incorporated in the model include real GDP which stands for economic prosperity, net inward FDI, industrialization (IND) measured by the total number of industries, coal consumption measured in metric tons, and total natural sources rent (TNR). This data is extraction of the World Bank database and is transformed into their natural log form to ascertain the growth level of the series. The functional model consists of five variables which include GDP, FDI, IND, coal consumption, and total natural resource rent (TNR) as explained above. It implies that economic growth (GDP) is the function of FDI inflow, industrialization (IND), coal consumption, and total natural resource rent (TNR).

These variables were carefully selected considering the significant role played by energy generated through coal usage to foster the economic fortune of South Africa. For instance, coal use generates the highest energy for power supply which by extension should promote the course of economic expansion because no sector of the economy could operate effectively and efficiently without power supply. Coal alone accounts for 95% of the total energy generated in South Africa World Bank development indicator (2007 and 2008). It implies that coal usage is a key factor in the equation of economic advancement in South Africa. This applies to the total natural resource rent (TNR) factor as the nation is known to be rich in terms of natural endowments such as platinum and goal which form the major export of the economy. Similarly, industrialization and FDI inflow are also a critical factor in the economic fortune of South Africa. South Africa has been the gateway of the inward FDI that flows to sub-Saharan Africa as well as second in Africa UNCTAD (2013). The report of the World Bank indicator (2018) shows that South Africa is ranked the 38th top in the world and second largest in Africa shore in terms of industrialization. These unique characteristics favor the enclosure of these variables in the model to undertake this study. Thus, the econometric model of the study is given as follows:

1 $$ RGDP=f\left( FDI, IND, COAL, TNR\right) $$

2 $$ LnGDP={\beta}_0+{\beta}_1 LnFDI+{\beta}_2 LnIND+{\beta}_3 LnCOAL+{\beta}_4 LnTNR+{\mu}_t $$

where the *B* represents the intercept of the model, while *B*_*1*_ and *B*_*2*_ are parameter estimates which stand for the elasticity value or coefficient of FDI and industrialization (IND).

### ARDL bounds testing to cointegration

The choice of this study to adopt the ARDL bound testing to cointegration as developed by Pesaran et al. (2001) became necessary because of its dynamic applicability irrespective of the nature of the integration of the series. This is the advantage that ARDL has over the traditional method in use. Pesaran et at. (2001) went further to note that another advantage of ARDL over other method is that it carries out a dual estimation; first, it estimates both the short- and long-run interaction between the variable of the functional model, and secondly, it investigates the causal effect existing between the series.

Thus, the formula is presented below:

3 $$ \varDelta Z={\mu}_0+{\mu}_1t+{\lambda}_1{\delta}_{t-1}+\sum \limits_{i-1}^k{\delta}_1{\nu}_{it-1}+\sum \limits_{j-1}^n{\varphi}_j\varDelta {Z}_{t-j}+\sum \limits_{i-1}^k\sum \limits_{j-1}^n{\phi}_{ij}\varDelta {V}_{it-j}+\varUpsilon {D}_t+{\mu}_t $$

where *v*_*t*_ estimate vector and *D* accounts for breakpoint as an exogenous variable. The hypothesis of the bound using *f*-statistic is stated below:

$$ {\displaystyle \begin{array}{l}{H}_0:{\delta}_1={\delta}_2=\dots .={\delta}_{K+2}=0\\ {}{H}_1:{\delta}_1\ne {\delta}_2\ne \dots .\ne {\delta}_{K+2}\ne 0\end{array}} $$

Thus, the rejection of *H*_0_ indicates evidence of long-run equilibrium between the series and revise is the case.

### Bayer and Hanck combined technique to cointegration

The econometrics literature has well-documented methodologies to cointegration relationship among variables of interest in the last decades. One of the most recent is the Bayer and Hanck (2013) methodology. The Bayer-Hanck (B-H) techniques have circumvented for the pitfalls of previously known single and multiple cointegration test (Engle and Granger 1987; Johansen and Juselius 1990; Phillips and Ouliaris 1990). The B-H test combined the individual statistics of Boswijk and Banerjee test and Johansen, Engle, and Granger test to form the B-H combined test statistics. This ability makes the test more powerful and robust, and estimates more reliable for decision framework. The B-H formula is presented below:

4 $$ EG- JOH=-2\left[\log \left({P}_{roEG}\right)+\left({P}_{roJOH}\right)\right] $$

5 $$ EG- JOH- BO- BDM=-2\left[\log \right(\left({P}_{roEG}\right)+\left({P}_{roJOH}\right)+\left({P}_{roBO}\right)+\left({P}_{roBDM}\right) $$

where, *P*_*roEG*_, *P*_*roJOH*_, *P*_*roBO*_*andP*_*roBDM*_ are the individual test probability test statistics as earlier mentioned. The null hypothesis of the B-H test is no cointegration. Thus, rejection of the null indicates presence of the equilibrium relationship among investigated series.

### Causality test

Granger (1969) attempted for the first time to determine the causal link between the series in a clear and simple term. Thus, a time series (*X*) is considered to granger cause another time series (*Y*) if the predicted error of current *Y* declines by using past values of *X* in addition to past values of *Y*. Conducting Granger causality with a non-stationary data could cause spurious regression, thus, resulting to a misleading conclusion. Stock and Watson (1988) assert that subjecting non-stationary series to causality test could lead to spurious regression for which the result could be misleading. Thus, going by the position of Engle and Granger (1987), this study adopted ADF and PP unit root tests for the series of interest which all series were confirmed to be stationary. Thus, the causal relationship of the series is conducted with all sense of accuracy. Similarly, the model was subjected to major diagnostic test such as a stability test by adopting the CUSUM and CUSUMSQ. Heteroscedasticity, normality and autocorrelation tests were estimated to confirm the robustness of the model all in an attempt to avoid the misleading result.

## Preliminary analysis

This section presents the preliminary test proceeding with the graph of the series indicating how the series are trended, followed by the summary statistic and correlation coefficient matrix. The summary statistic in Table 1 proved that industrialization has a larger average comparatively. The level of dispersion of the variables from their mean is empirically evidently as indicated by their standard deviation. The Jarque-Bera indicates a scenario of the overall normal distribution of the variables under study. Furthermore, Table 2 presents the correlation coefficient matrix results which show, on the average, a strong correlation between the series of interest. Interestingly, the two strongest connections exist between industrialization and economic prosperity, and between coal usage and industrialization, which is in line with economic intuition as expected Table 3.

**Table 1:** Summary statistics of underlined variables

	LNGDP	LNFDI	LNIND	LNCOAL	LNTNR
Mean	8.7815	− 0.6769	25.1698	4.1675	1.5830
Median	8.7753	− 0.5269	25.1288	4.2827	1.5888
Maximum	8.9336	1.7882	25.4077	4.5414	2.5600
Minimum	8.6157	− 5.9931	24.9116	3.3088	0.6503
Std. dev.	0.1034	1.5855	0.1622	0.3701	0.4494
Skewness	0.0573	− 1.3647	0.0659	− 1.2052	0.1585
Kurtosis	1.7558	5.2970	1.7768	3.1228	2.6974
Jarque-Bera	2.5369	20.6790	2.4596	9.4664	0.3121
Probability	0.2812	0.0000	0.2923	0.0088	0.8555
Sum	342.4798	− 26.4001	981.6241	162.5335	61.7407
Sum sq. dev.	0.4066	95.5272	0.9995	5.2047	7.6734
Observations	39	39	39	39	39

Variables are in their natural log form

**Table 2:** Correlation coefficient matrix analysis

Observations	GDP	FDI	IND	COAL	TNR
GDP	1.000				
FDI	0.229	1.000			
IND	0.756***	0.351**	1.000		
COAL	0.369***	0.348**	0.864***	1.000	
TNR	0.452***	− 0.078	0.259**	0.099	1.000

Series are in their level form

**Table 3:** Non-stationary results

Level form	LNGDP	LNFDI	LNIND	LNCOAL	LNTNR
τ_T_ (ADF)	− 1.384	− 3.906**	−1.551	− 1.039	− 2.834
τ_μ_ (ADF)	0.874	− 3.575**	− 0.344	− 3.165**	− 2.955**
τ (ADF)	0.599	3.303***	2.968	2.871	− 0.253
τ_T_ (PP)	− 1.073	− 3.781**	− 1.763	− 0.943	− 2.774
τ_μ_ (PP)	0.605	− 3.475**	− 0.394	− 3.321**	− 2.927**
τ (PP)	0.855	− 3.100***	2.796	2.551	− 0.098
First difference	LNGDP	LNFDI	LNFDI	LNURB	LNCOAL
τ_T_ (ADF)	− 4.355***	− 4.524***	− 5.837***	− 6.867***	− 0.8.139***
τ_μ_ (ADF)	− 4.265***	− 8.182***	− 5.899***	− 5.934***	− 8.121***
τ (ADF)	− 4.253***	− 8.305***	− 5.156***	− 5.185***	− 8.185***
τ_T_ (PP)	− 4.301***	− 8.458***	− 5.839***	− 6.903***	− 8.139***
τ_μ_ (PP)	− 4.258***	− 8.606***	− 5.902***	− 5.934***	− 8.143***
τ (PP)	− 4.243***	− 8.706***	− 5.215***	− 5.205***	− 8.208***

***Significance at 0.01

**Significance at 0.05

**Fig. 2:**
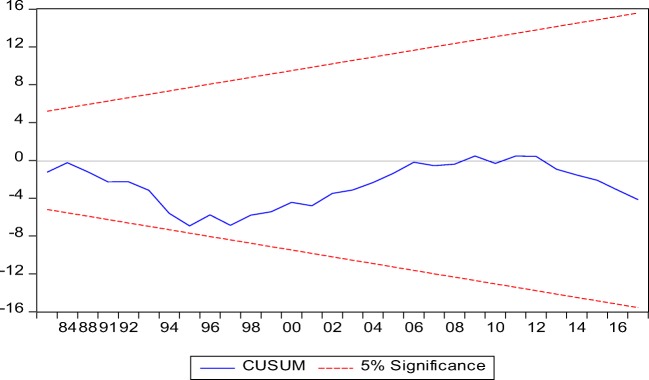
CUSUM

**Fig. 3:**
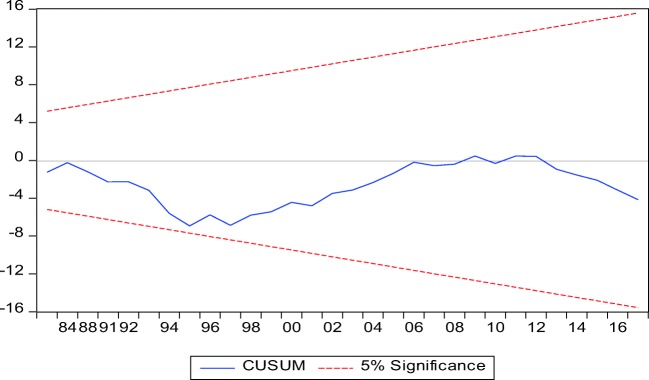
CUSUMSQ

Table 4 presents the results from the ADF and PP unit root tests, which shows that series were stationary at level except for industrialization and GDP. FDI is stationary at level. However, stationarity was established at 1% degree of freedom for all variables after the series were differenced at first instanced for both ADF and PP tests. Thus, the overall outcome signified a mixed order of stationarity which informed the study to adopt the ARDL bound test as the most suitable method. The model was found to homoscedastic in nature with no case of serial correlation. The Ramsey reset test confirmed the validity of the model specification. While results from CUSUM and CUSUMSQ presented in Figs. 2 and 3 show the stability of the functional model since the blue line falls within the accepted margin of 5% level of significance (Okunola 2016).

**Table 4:** ARDL long- and short-run result. Model: RGDP = *f* (FDI, IND, COAL, TNR)

Variables	Coefficient	SE	*t*-statistic	*P* value
Short run				
FDI	0.002	0.002	1.027	0.317
IND	0.506***	0.075	6.789	0.000
COAL	− 0.083***	0.024	− 3.371	0.003
TNR	0.019**	0.009	2.042	0.055
ECT	− 0.39***	0.042	− 9.569	0.000
Long run				
FDI	0.013**	0.006	2.101	0.049
IND	1.274***	0.159	8.039	0.000
COAL	− 0.207***	0.046	− 4.524	0.000
TNR	0.046	0.021	2.226	0.038
Diagnostic tests				
Tests	*f*-statistic	Prob. value		
*χ*^2^ SERIAL	0.354	0.705	*f* (2,28)	
*χ*^2^ WHITE	1.693	0.181	*f* (26,11)	
*χ*^2^ RAMSEY	1.357	0.254	*f* (1,29)	

*1% level

**5% level

***10% level

## Empirical interpretation and discussion

This section presents the main empirical result starting with the dynamic ARDL bounds test presented in Table 4 which reveals that FDI inflow influences economic growth positively in an insignificant way in the short run. But the tight changes in the long run where FDI exerts a positive and significant impact on economic prosperity. Statistically, a 1% change in FDI accounts for about 0.002% and 0.001% transformation in economic expansion in the short-long distance. This implies that the benefits of the investor flowing into the economy will be realized only in the long after successful conversion of their potential into economic gain which should be noted by the authority concern.

Industrialization, on the other hand, demonstrates a positive and significant impact on the growth process both in the short and long run. Industrialization contributes 0.506% and 1.274% to economic expansion both in the short and long run respectively as consequences of its 1% change. This implies that embarking on industrial development will not just produce an immediate positive benefit to economic prosperity but also serve as a strong pillar for achieving economic expansion in the future. The government of South African should strive to partner with the private sectors to set the nation on the fast track of industrialization as one sure way of achieving economic expansion.

Furthermore, the findings prove that coal consumption exerts a significant negative impact on GDP both in the present and future term. A per-cent increase in the consumption of coal will influence GDP negatively by 0.083% and 0.207%. This is not surprising as coal usage empirically proves to be an emitter of carbon which could be harmful to the environment and by extension to the economic expansion. Similarly, TNR exhibit positive and significant influence on economic prosperity both in the short run and in the distance future accounting for about 0.02% and 0.05% respectively. This is consistent with our expectation as natural deposits in South Africa are a formidable force behind the economic fortune of the country. South Africa economy is highly rich ranking top in natural resources in Africa, notable among them are platinum and goal. Thus, the gains from these deposits must be injected particularly into the productive sector to help generates economic expansion. Finally, results further show that it takes about 39% for the GDP to adjust to a stable point every year through the influence of the regressors. Finally, Table 5 renders the recent and novel Bayer and Hanck (2013) combined cointegration test, rejecting the null of no cointegration. Similarly, the ARDL bound tests from *f*-stat and *t*-bound tests as presented in Table 6 show that we reject the null hypothesis at 10, 5, 2.5, and 1% respectively and conclude that the variables converge in the long run.

**Table 5:** Bayer and Hanck result to cointegration

Fitted model	EG-JOH	EG-JOH-BO-BDM	Cointegration remark	
LnGDP = *f* (LnFDI,LnIND,LnCOAL,LNTRN)	68.464**	35.988	Yes	
Critical values	29.444	19.878	Yes	
ARDL bounds test results to cointegration				
*f*-bounds test	Null hypothesis: no levels relationship			
Test statistic	Value	Sig.	I(0)	I(1)
*f-*statistic	15.131*	10%	3.03	4.06
*K*	4	5%	3.47	4.57
		2.5%	3.89	5.07
		1%	4.4	5.72
*t*-bounds Test	Null hypothesis: no levels relationship			
Test statistic	Value	Sig.	I(0)	I(1)
*t*-statistic	− 9.569	10%	− 3.13	− 4.04
		5%	− 3.41	− 4.36
		2.5%	− 3.65	− 4.62
		1%	− 3.96	− 4.96

Source**:** author computation

**Table 6:** Granger block exogeneity results

Excluded	Chi square	df	Prob
Dependent variable: RGDP			
LNFDI	5.522**	1	0.0188
LNIND	0.498	1	0.4806
LNCOAL	3.623**	1	0.0570
LNTNR	16.479***	1	0.0000
All	68.697***	4	0.0000
Dependent variable: LNFDI			
LNGDP	0.5782	1	0.4470
LNIND	0.809	1	0.3682
LNCOAL	5.763**	1	0.0164
LNTNR	4.532**	1	0.0333
All	17.955***	4	0.0013
Dependent variable: LNIND			
LNGDP	0.831	1	0.3619
LNFDI	4.470**	1	0.0345
LNCOAL	0.375	1	0.5402
LNTNR	13.188***	1	0.0003
All	48.829***	4	0.0000
Dependent variable: LNCOAL			
LNGDP	1.398	1	0.2370
LNFDI	9.364***	1	0.0022
LNIND	4.334**	1	0.0374
LNTNR	3.394**	1	0.0654
All	292.542***	4	0.0000
Dependent variable: LNTNR			
LNGDP	1.516	1	0.2183
LNFDI	4.374**	1	0.0365
LNIND	0.896	1	0.3438
LNCOAL	0.053	1	0.8176
All	145.396***	4	0.0000

***Significance at 0.01

**Significance at 0.05

Table 6 represents the result from Granger causality test which indicates a unidirectional link between FDI inflow and economic prosperity confirming the FDI induced economic expansion hypothesis as supported by the work of Sarkodie and Strezov (2019), Pradhan et al. (2019), and Tshepo (2014). This implies that FDI inflow is a key player in the growth equation of South Africa; thus, the authority concern should carefully consider putting in place macroeconomic measures such as tax exemption, a free license that would woo new investor into the economy. The result reveals a striking outcome, a non-causal relationship between industrialization and economic expansion.

The empirical finding further reveals a one-way link only from coal consumption to economic expansion consistent with growth hypothesis and some empirical studies (Bekun et al. 2019a, b; Wolde-Rufael 2009). It is worth noting that conservation policy will be harmful to the economic expansion of South Africa. Instead, a well-articulated coal-intensive policy which encourages the consumption of coal as an alternative source of energy will benefit and set the economy on a path to achieving quick economic advancement. Interestingly, the findings prove that only TNR drives GDP following our a priori expectation. This suggests that natural endowments in South Africa are a blessing and act as an agent of economic transformation rather than a curse. The authority concern should carefully indulge in harnessing the natural deposits in the nation and to make judicious use of the earnings thereof to improve economic expansion which by extension should better the lives of the citizenry.

Another outcome shows a two-way interaction between FDI inflow and coal consumption. Coal consumption could influence FDI indirectly in the form of power generation and supply to power the smooth operation of foreign firms. On the other hand, foreign firms could trigger the derived demand for coal consumption through energy demand. Similarly, a two-way causal effect exists between FDI inflow and TNR in line with our initial expectation. This suggests that the natural riches of South Africa such as platinum and goal are the strong determinants of FDI that flow into the economy. Claassen et al. (2011) and Carike et al. (2012) both posit that in the developing economies particularly Africa, most of the FDI flows in in the form of technological diffusion to harness the natural endowments.

Further findings prove a one-way causal link running only from FDI to industrialization contradicting our a priori expectation and are supported by past studies (Nunnenkamp and Spatz 2003, Singh and Jun 1999). This suggests that the condition of local industry in South Africa does not play any role in wooing investor into the economy, rather the spillover influence of FDI inflow such as technological diffusion help drive the local industries to maturity. According to the findings from this study, what determines the inflow of FDI into the country is natural resources and coal consumption which is educative to the authority concern.

A one-way link exists from industrialization to coal consumption which implies that the operation of the industrial sector causes an increase in the derived demand for coal consumption through the demand for power supply. The more industry expands the more demand for the power supply, thus, increase demand for coal usage. This is true because coal is the largest generator of energy in South Africa. This is similar to the case of TNR which drive coal consumption without a feedback reaction. Normally, natural resources are harnessed by different companies that consume energy to generate power for their smooth operation. The functional operation of the companies to harness the natural endowment of the nation influences derived demand for coal consumption through increased demand for energy. Finally, only FDI inflow drives TNR which is in line with our expectation. FDI inflow promotes natural deposits through its technological diffusion. This so-called foreign superior technology is used in harnessing the natural riches for which the proxy is spent to sponsor the course of economic expansion.

## Conclusion

The connection between inflow of Foreign Direct Investment into South Africa and the prosperity of the economy has been an issue of increasing concern with past studies revealing either a positive or negative connection with no consensus in the debate. Given the foregoing, this study mainly seeks to investigate FDI-led growth hypothesis with a specific emphasis on the role of industrialization in attraction FDI inflow. The results have confirmed the said hypothesis went further to prove that industrialization is not a determining factor in attracting FDI inflow into South Africa. Rather, the key players in attracting FDI inflow are coal consumption and natural deposits of the nation. This is an indication that FDI inflow into the economy is a key fact for economic advancement. The second implication is that improving the condition of the local industries is not a pre-condition to attracting new potential investors into the economy neither does it develop the absorptive capacity of the economy as argued in the literature (Nunnenkamp and Spatz 2003; Singh and Jun 1999).

The unidirectional interaction from coal consumption to economic growth implies that coal-intensive policy should be chosen in place of conservative policy which is very instructive to the South African (SA) policy makers. However, coal is not a clean energy source. Thus, the need for a paradigm shift to cleaner energy sources and technologies in her energy mix is encouraged, which are reputed to be more environmentally friendly for the SA economy (Emir and Bekun 2019). In summary, to set the economy on the right path for achieving economic prosperity, policy makers should develop the absorptive capacity of the economy to attract new investors, coupled with the creation of a stable macroeconomic environment and the implementation of tax exemption or tax holidays to encourage an inflow of new investors to the energy sector. This could be complemented by economic growth policy that encourages increase consumption of coal as an alternatives source of energy which is cheaper comparatively. Finally, the course of economic prosperity could be achieved through proper investment of the gain from the natural deposits of the nation. The government should ensure that the revenue from the natural endowment is injected into the productive sector of the economy to ensure maximum return that will transcend to economic expansion.
